# Inhibition of cholesterol metabolism underlies synergy between mTOR pathway inhibition and chloroquine in bladder cancer cells

**DOI:** 10.1038/onc.2015.511

**Published:** 2016-02-08

**Authors:** M A King, I G Ganley, V Flemington

**Affiliations:** 1 AstraZeneca Oncology, Alderley Park, Macclesfield, Cheshire, UK; 2 MRC Protein Phosphorylation and Ubiquitylation Unit, University of Dundee, Dundee, UK; 3 AstraZeneca Oncology, CRUK Cambridge Institute, Li Ka Shing Centre, Cambridge CB2 0RE, UK

## Abstract

Mutations to fibroblast growth factor receptor 3 (FGFR3) and phosphatase and tensin homologue (PTEN) signalling pathway components (for example, PTEN loss, PIK3CA, AKT1, TSC1/2) are common in bladder cancer, yet small-molecule inhibitors of these nodes (FGFR/PTENi) show only modest activity in preclinical models. As activation of autophagy is proposed to promote survival under FGFR/PTENi, we have investigated this relationship in a panel of 18 genetically diverse bladder cell lines. We found that autophagy inhibition does not sensitise bladder cell lines to FGFR/PTENi, but newly identify an autophagy-independent cell death synergy in FGFR3-mutant cell lines between mTOR (mammalian target of rapamycin) pathway inhibitors and chloroquine (CQ)—an anti-malarial drug used as a cancer therapy adjuvant in over 30 clinical trials. The mechanism of synergy is consistent with lysosomal cell death (LCD), including cathepsin-driven caspase activation, and correlates with suppression of cSREBP1 and cholesterol biosynthesis in sensitive cell lines. Remarkably, loss of viability can be rescued by saturating cellular membranes with cholesterol or recapitulated by statin-mediated inhibition, or small interfering RNA knockdown, of enzymes regulating cholesterol metabolism. Modulation of CQ-induced cell death by atorvastatin and cholesterol is reproduced across numerous cell lines, confirming a novel and fundamental role for cholesterol biosynthesis in regulating LCD. Thus, we have catalogued the molecular events underlying cell death induced by CQ in combination with an anticancer therapeutic. Moreover, by revealing a hitherto unknown aspect of lysosomal biology under stress, we propose that suppression of cholesterol metabolism in cancer cells should elicit synergy with CQ and define a novel approach to future cancer treatments.

## Introduction

Bladder cancer has a worldwide incidence of roughly 400 000 cases and 150 000 deaths per year, yet there are currently no targeted therapeutics available to patients.^1^ The disease is genetically complex and presents with a predominance of activating mutations to fibroblast growth factor receptor 3 (FGFR3) and the phosphatidylinositol 3-kinase/AKT/mammalian target of rapamycin (PI3K/AKT/mTOR) (phosphatase and tensin homologue (PTEN)) pathway, highlighting therapeutic opportunities at these nodes.^1, 2, 3, 4^ Nevertheless, small-molecule inhibitors to these kinases have so far proven ineffective in preclinical models and there is considerable interest in determining the modes of resistance to FGFR and PTEN pathway inhibitors (FGFR/PTENi) in bladder cancer.

There is a direct and well-characterised link between AKT/mTOR signalling and macroautophagy (autophagy), which may promote cancer cell survival under PTEN pathway inhibition.^5, 6, 7, 8, 9^ Specifically, the efficacy of AKT inhibitors in bladder and prostate cancer models, and EGFR/HER2 inhibition in breast and lung carcinomas, is promoted by inhibiting autophagy with chloroquine (CQ).^10, 11, 12, 13^ Autophagy describes the bulk sequestration of cytosol into double-membraned vesicles and its fusion to the lysosome, wherein substrates are degraded and recycled to support homeostasis under stress.^14^ Under normal conditions, mTOR represses autophagy via the ATG13/ULK/FIP200 complex, which in turn directs the nucleation of autophagosomes through the Beclin/VPS34 complex.^15, 16, 17, 18^ Following activation of autophagy, two ubiquitin-like systems regulated by ATG7 mediate autophagosome maturation and completion by LC3 lipidation.^19, 20, 21^

We have investigated a function for autophagy in a panel of 18 bladder cancer cell lines treated with small-molecule inhibitors targeting nodes of potential therapeutic relevance: FGFR, PI3Kβ/δ, AKT and mTOR (FGFR/PTENi). Modulation of cell death was quantified under these conditions and a function for autophagy was assayed by knockdown of multiple essential components (ATG13, ULK1/2, VPS34, ATG7, ATG3, ATG16L1 and ATG14), CRISPR/Cas9 *ATG7* knockout (KO) and chemical inhibitors including CQ, bafilomycin A1 (BafA1) and 3-methyladenine (3MA). Our data reveal little evidence for autophagy in the promotion of survival under FGFR/PTENi, but highlight an autophagy-independent synergistic cell death between AKT or mTOR inhibition and CQ in FGFR-dependent cell lines.

Synergistic cell death showed features of lysosome-initiated apoptosis, including cathepsin-dependent caspase activation. We show that inhibition of FGFR/PI3Kα/AKT/mTOR nodes suppress the expression of enzymes regulating cholesterol metabolism in FGFR-dependent cell lines, correlating with the degree to which these compounds potentiate CQ-induced cell death. This form of CQ-driven synergistic cell death is profoundly inhibited by saturating cellular membranes with cholesterol, or recapitulated by inhibiting cholesterol metabolism with atorvastatin (Ato) and knockdown of cholesterol biosynthesis enzymes. Moreover, we found that all FGFR3-mutant cell lines regulate cSREBP1 expression in an mTORC1/2-dependent manner, sensitising these—but not FGFR-wild type (FGFR-WT)—cell lines to CQ-induced cell death under mTORi. These results elucidate how CQ, which is currently being used as a cancer therapy adjuvant in over 30 clinical trials, synergises with inhibitors of mTOR signalling and defines a patient cohort predicted to respond to this combination. This work also highlights a fundamental feature of lysosomal membrane biology, suggesting that cancer therapeutics that impact cholesterol metabolism should combine with CQ and provide an innovative approach to cancer treatment.

## Results

### Genetic inhibition of autophagy does not increase the sensitivity of bladder cancer cell lines to FGFR/PTENi

**Figure 1:**
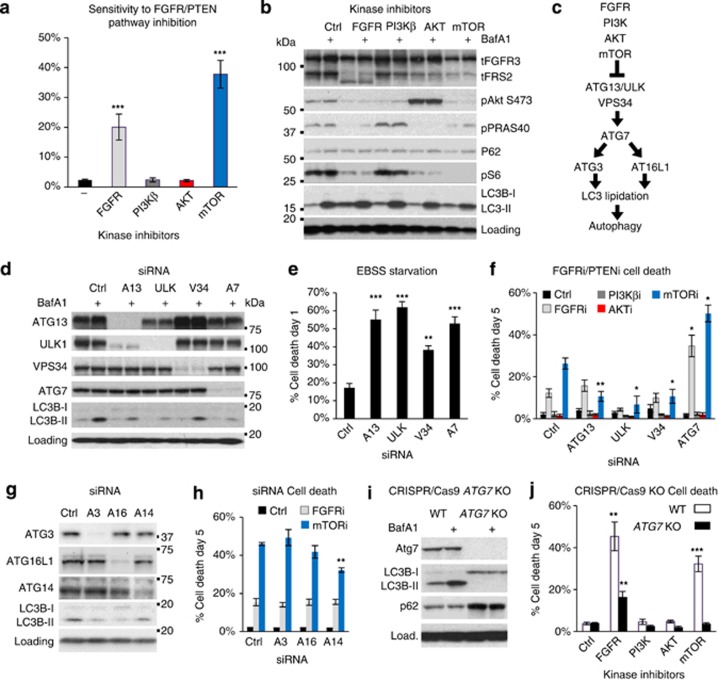
Activation of autophagy does not contribute to survival under FGFR/PTENi. (**a**) RT112 cells were treated with the indicated inhibitors at 1 μm for 5 days and assayed for cell death. Histogram shows the number of dead cells (SYTOX Green-positive) as a proportion of total cell number (Hoechst-positive; *n*⩾7; mean±s.e.). (**b**) RT112 were incubated with kinase inhibitors for 22 h before the addition of BafA1 (20 nm), where indicated, for the last 2 h. A representative immunoblot confirms target engagement for FGFRi (FRS-2 bandshift), AKTi (hyperphosphorylated in catalytically inactive state), mTORi (S6 dephosphorylation) and BafA1 (LC3B; *n*=3). (**c**) Schematic to illustrate mTOR regulation of autophagy. (**d**–**h**) RT112 cultures were reverse transfected with the indicated autophagy-targeting siRNAs (10 nm) for 48 h before analysis. (**d**) Efficiency of protein knockdown and inhibition of autophagic LC3 flux (−/+BafA1) was assayed by immunoblotting at 72 h (*n*=3). (**e** and **f**) Histograms show the quantification of cell death in cultures starved of amino acids and serum (EBSS) for 24 h (**e**; *n*=5; mean±s.e.) or treated with the indicated kinase inhibitors for 5 days (*n*=4; mean±s.e.). (**g**) Immunoblot confirms knockdown of the indicated autophagy-essential proteins and inhibition of LC3 lipidation. (**h**) Histogram shows quantification of cell death after treatment with FGFRi or mTORi for 5 days (*n*=3; mean±s.e.). (**i**) An *ATG7* KO cell line was engineered by CRISPR/Cas9 and immunoblotted to confirm loss of ATG7 expression and LC3 lipidation. (**j**) *ATG7* KO RT112 cultures were assayed for cell death as in (**a**) (*n*=4; mean±s.e.). *P<0.05, **P<0.01, ***P<0.001.

Autophagy is reported to promote survival under RTK and AKT inhibition in multiple preclinical cancer models. We therefore used a selection of highly selective and potent kinase inhibitors targeting relevant genetic aberrations along these pathways in bladder cancer cell lines: FGFRi (AZD4547), PI3Kβ/δi (AZD8186), AKTi (AZD5363) and mTORi (AZD2014); and assayed cell death over 5 days.^22, 23, 24, 25^ Data relating to RT112, which express constitutively active FGFR3 (FGFR3-TACC3), are shown in Figure 1 and used throughout this study to represent our major findings.^26^ RT112 cultures showed marked sensitivity to both FGFRi and mTORi, but tolerated AKTi and PI3Kβi (Figure 1a). This pattern of cell death was also observed in MGH-U3—defining these two cell lines as FGFR-dependent—but absent across all other cell lines (Supplementary Figure S1A). Nevertheless, FGFRi significantly reduced proliferation in all FGFR3-mutant cell lines, whereas mTORi reduced proliferation across the panel (Supplementary Figure S1C). Western blotting confirms target engagement, also showing that chronic AKT/mTOR inhibition increases the efficiency of LC3 lipidation, but suppresses the volume of LC3 flux (Figure 1b and Supplementary Figures S1B–D).^27^

The contribution of autophagy to survival under FGFR/PTENi was assayed by small interfering RNA (siRNA)-mediated knockdown of key signalling components coupling mTOR to autophagy activation: ATG13, ULK1/2 and VPS34; and the core autophagy protein ATG7 (Figure 1c). Considerable suppression of protein expression and autophagic flux was achieved in RT112 cells (Figure 1d), and validated across six bladder cancer cell lines (ATG13: 95% knockdown, 68% flux inhibition; ULK: 97% knockdown, 61% flux inhibition; VPS34: 94% knockdown, 56% flux inhibition; and ATG7: 96% knockdown, 51% flux inhibition). A functional consequence of autophagy inhibition by siRNA is demonstrated by the exacerbation of cell death following starvation in Earle's balanced salt solution (EBSS) for 24 h (Figure 1e). Importantly, in combination with FGFR/PTENi, inhibiting the activation of autophagy (ATG13, ULK and VPS34 siRNAs) did not increase cell death in RT112 or any other bladder cell line, irrespective of genetic aberrations to the PTEN pathway, although proliferation was moderately suppressed (Figure 1f and Supplementary Figures S2A–C).

RT112 cells showed some evidence for the potentiation of FGFR/mTORi-induced cell death by ATG7 siRNA (Figure 1f); however, on deconvolving the SMARTpool, we found significant variability in cytotoxicity despite similar ATG7 knockdown (Supplementary Figure S2D). We therefore assayed the role of core autophagy components downstream of ATG7 by siRNA knockdown of ATG3, ATG16L1 and ATG14. Again, however, we show that these components do not contribute to the innate resistance of RT112 cells to FGFR/PTENi (Figures 1g and h). To assay directly a function for ATG7, we generated *ATG7* KO clones from RT112 cultures using CRISPR/Cas9. RT112 *ATG7* KO cultures were disabled for LC3 lipidation and autophagy (Figure 1i), yet showed no increase in sensitivity to FGFR/PTENi (Figure 1j). Conversely, *ATG7* KO cells showed increased tolerance for FGFRi and mTORi, similar to that observed under ATG13, ULK and VPS34 knockdown (Figure 1f). Thus, neither the activation of autophagy nor core autophagic processes contribute to the innate resistance of bladder cancer cell lines to FGFR/PTENi.

### Potentiation of CQ-induced cell death by AKTi is limited to FGFR-dependent cell lines

**Figure 2:**
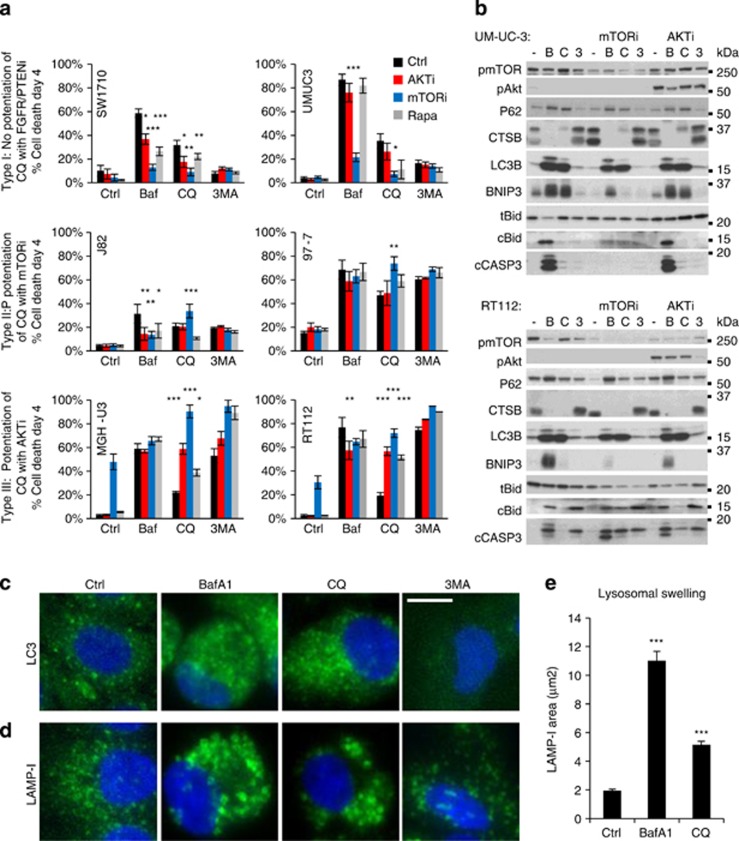
Potentiation of CQ-induced cell death by AKTi or mTORi is restricted to FGFR3-dependent cell lines. Bladder cancer cell lines were preincubated with AKTi (1 μm), mTORi (1 μm) or Rapa (100 nm) for 30 min before the addition of BafA1 (Baf; 20 nm), CQ (20 μm) or 3MA (10 mm). (**a**) Histograms show quantification of cell death at day 4 in two representative cell lines of the three major response types described in the text (*n*=4; mean±s.e.). (**b**) UM-UC-3 and RT112 cells were treated as in (**a**), lysed and immunoblotted for the indicated autophagic, lysosomal and apoptotic proteins. (**c** and **d**) Immunocytochemistry for LC3 (**c**) and LAMP-I (**d**) in RT112 cells treated as in (**a**) reveals autophagosome accumulation (**c**) and lysosomal swelling (**d**), quantified using ImageJ in (**e**; mean±s.e.; *n*=3). *P<0.05, **P<0.01, ***P<0.001.

As previous studies reported synergistic cell death between CQ and AKTi, we next screened the bladder panel for combinations of chemical autophagy inhibitors and AKTi or mTORi. CQ, BafA1 and 3MA were used to block autophagy, whereas rapamycin (Rapa) was included to inhibit mTORC1. Over 4 days, a clear difference emerged in the pattern of cell death across the bladder panel, dividing the cell lines into three types (Figure 2a and Supplementary Figure S3). Type-I cells displayed no evidence for the potentiation of CQ-, BafA1- or 3MA-induced cell death with AKT or mTOR inhibitors. Conversely, most lines showed significant protection against BafA1 or CQ cytotoxicity with mTORi (for example, UM-UC-3 and SW1710), correlating with an inhibition of BNIP3 induction, BID cleavage and caspase-3 (CASP3) activation (Figure 2b). This protection was recapitulated by blocking cell cycle progression with the CDK4/6 inhibitor palbociclib, indicating that BafA1-induced cell death is compounded by proliferation (Supplementary Figures S3B and C). Type-II cells are defined by the moderate potentiation of CQ-induced cell death with mTORi, all of which express activating FGFR3 mutations (for example, J82, 97-7). Finally, type-III cells show substantial potentiation of CQ-induced cell death with both AKT and mTOR inhibitors. This response was only observed in FGFR-dependent cell lines—MGH-U3 and RT112—and correlated with BID cleavage and caspase-3 activation (Figures 2a and b). These cells also show moderate potentiation of 3MA-induced cell death with AKTi and mTORi; however, when compared with the exquisite selectivity of BafA1, it is unlikely that this effect is related to inhibition of autophagic flux (Supplementary Figures S3F and G). Rapa treatment demonstrates that type-III cells tolerate mTORC1 inhibition alone, but that targeting this node is sufficient to potentiate cell death with CQ. In contrast, CQ-induced cell death is significantly abated by palbociclib, indicating that similar to BafA1, proliferation exacerbates CQ toxicity (Supplementary Figure S3H).

Disruption to autophagy by BafA1 and CQ is confirmed by the accumulation of LC3-II by immunoblotting and immunocytochemistry, revealing that lysosomes swell to 2–5 times their original size under these conditions (Figures 2b–d). In contrast, 3MA showed little effect on LC3-II by immunoblotting, which is reported for other VPS34 inhibitors, yet prevented LC3 punctation by immunocytochemistry.^28^ Overall, this screen shows that chemical autophagy inhibitors do not generally increase the sensitivity of bladder cell lines to AKT or mTOR inhibitors, yet highlights an intriguing response to these compounds in combination with CQ in FGFR-dependent cells.

### AKTi and mTORi enhance CQ-induced LCD

**Figure 3:**
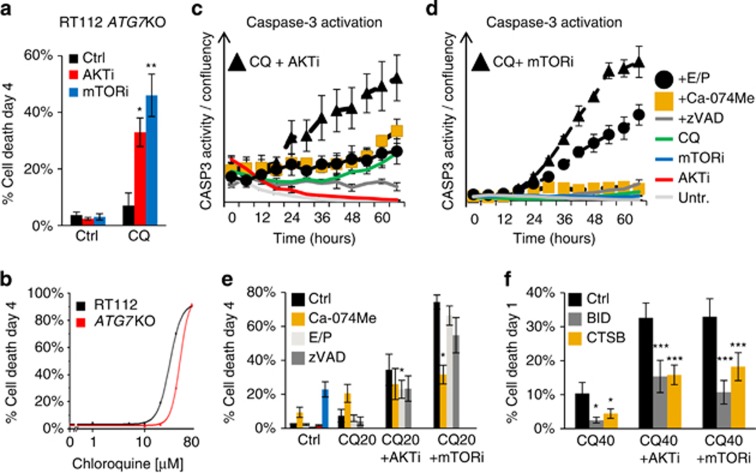
Synergy between CQ and AKTi or mTORi is autophagy-independent and driven by cathepsins. RT112 *ATG7* KO cells were incubated with AKT/mTORi (1 μm) before the addition of CQ (20 μm). (**a**) Histogram shows quantification of cell death at day 4, revealing synergy in autophagy-disabled cells (*n*=4; mean±s.e.). (**b**) RT112 WT and *ATG7* KO cells were incubated with CQ (1–80 μm) for 4 days and quantified for cell death. Line graph shows OriginLab curve fitting from nine independent experiments, from which cdEC50 and statistics (see text) were calculated. (**c** and **d**) RT112 cells were treated with the AKTi (**c**) or mTORi (**d**) and CQ (20 μm) in combination with Ca-074Me (10 μm), E64D and pepstatinA (E/P; 30 μm/30 μm) and zVAD (20 μm) for 3 days. Cultures were incubated with the caspase-3 fluorogenic substrate NucView-488 and imaged every 6 h for fluorescence (*λ*475(40)/535(45)) and cellular confluency. Data are plotted as the number of fluorescent nuclei divided by cellular confluence (data from one experiment is shown, representative of three independent experiments; mean±range). (**e**) RT112 cells were treated as in (**c** and **d**) for 4 days and assayed for cell death (*n*=3; mean±s.d.). (**f**) RT112 cells were reverse transfected with Ctrl, BID or CTSB siRNA for 48 h before treatment with CQ (40 μm). Histogram shows cell death quantification at 24 h (*n*=3; mean±s.e.). *P<0.05, **P<0.01, ***P<0.001.

We reproduced the potentiation of CQ-induced cell death by AKTi and mTORi in RT112 *ATG7* KO cells, to demonstrate conclusively that autophagy is unrelated to this cytotoxicity (Figure 3a). Although RT112 *ATG7* KO cells showed a small increase in CQ tolerance (cell death EC50 (cdEC50)=48 μm; *P*=0.003) versus wild-type (WT) (cdEC50)=30 μm), their ultimate sensitivity also confirms that CQ-induced cell death is autophagy-independent. CQ is known to cause lysosomal membrane permeabilisation, cathepsin leakage and activation of BID and caspases.^29, 30^ We therefore investigated the role of cathepsins in caspase activation and cell death induced by CQ in combination with AKTi and mTORi. We find that CASP3 activity is only observed when AKTi or mTORi are combined with CQ, whereas treatment with AKTi, mTORi or CQ alone is not sufficient to induce its activation (Figures 3c and d). Moreover, CASP3 activation under these conditions is completely suppressed by co-treatment with a cathepsin B/L inhibitor (Ca-074Me) and partially suppressed by the pancathepsin inhibitors E64D and pepstatinA.^31^ This assay clearly positions cathepsins upstream of caspase activation in this mode of cell death.

Cathepsin inhibition by Ca-074Me also considerably inhibited RT112 cell death with CQ and mTORi (from 74%, ±s.e. 4% to 31%, ±s.e. 6% *P*=0.004; Figure 3e), supporting the relevance of cathepsin-mediated caspase activation to cell death. Additionally, both Ca-074Me and the pancaspase inhibitor zVAD rescued the number of viable cells under mTORi+CQ treatment to roughly the same value as that measured for mTORi treatment alone: from 35% (±s.e. 13%) to 96% (±s.d. 13%) with Ca-074Me and to 84% (±s.d. 28%) with zVAD. That E64D and pepstatinA did not robustly protect against cell death or CASP3 activation under mTORi-induced potentiation of CQ toxicity is likely due to the hydrolysis of E64d—which inhibits cathepsins B, H and L—in the culture medium.^31, 32, 33^ To assay directly the role of CTSB in this model of cell death and clarify the involvement of BID, we knocked down these components by siRNA. Figure 3f shows that both BID and CTSB siRNA robustly inhibited cell death induced by CQ in combination with both AKTi and mTORi. The mechanism of synergistic cell death is therefore autophagy-independent, showing characteristics of apoptosis cross-talk activated by lysosomal membrane permeabilisation.^29^

### Suppression of cholesterol biosynthesis by AKTi and mTORi underlies synergy with CQ

Given that AKTi and mTORi only potentiate CQ-induced cell death in FGFR-dependent cell lines, we investigated how FGFR3 signalling might regulate lysosomal cell death (LCD). A possible link between these two pathways was found in reports showing that (i) FGFR3 maintains the expression of genes required for fatty acid and cholesterol biosynthesis via SREBP1; and (ii) lysosomal cholesterol can regulate membrane integrity and LCD.^34, 35^ FGFRi is known to induce cell death by suppressing SCD1 (stearoyl-CoA desaturase-13) expression, which we now found to also underlie mTORi cytotoxicity. Both FGFRi- and mTORi-induced cell death is rescued by oleic acid, the product of SCD1 activity (Supplementary Figure S4A), and correlates with complete suppression of SCD1 expression. However, as a lower dose of mTORi (0.25 μm) does not induce cell death, but potentiates CQ-induced cell death, we used this concentration for subsequent experiments.^34^

**Figure 4:**
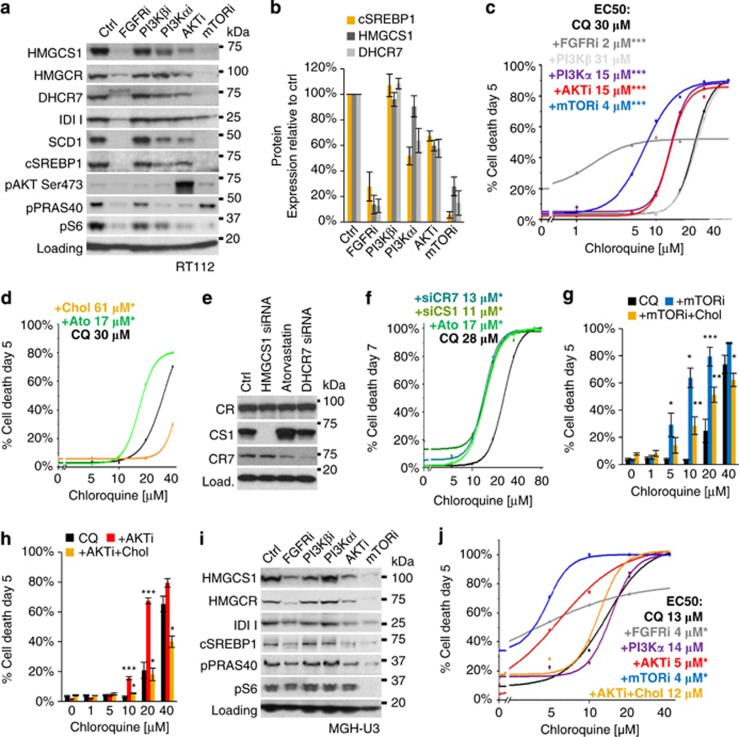
Inhibition of cholesterol metabolism underlies synergy between CQ and AKTi or mTORi. (**a**) RT112 were treated with FGFR/PTENi for 3 days and immunoblotted for the expression of enzymes regulating cholesterol and fatty acid biosynthesis pathways and quantified in (**b**) (*n*=3; mean±s.e.). (**c**) RT112 cells were treated with inhibitors to FGFR (0.25 μm), AKT (1 μm) and mTOR (0.25 μm) for 5 days in combination with CQ (1–40 μm). Line graph shows OriginLab curve fitting based on data points generated in >3 independent experiments. Inset text shows EC50 values and statistical analysis for each treatment. (**d**) RT112 cells were preincubated with Ato (5 μm) or water-soluble cholesterol (Chol, 10 μg/ml) for 6 h before the addition of CQ (1–80 μm). Line graphs were curve-fitted using cell death data from three independent experiments at each data point. (**e**) RT112 cultures were reverse transfected with siRNA targeting HMGCS1 or DHCR7 for 72 h or Ctrl siRNA and incubated with or without Ato for 24 h. Immunoblotting shows expected protein knockdown by siRNA (*n*=2). (**f**) Cells were transfected as in (**e**) and treated with CQ (1–80 μm) from days 2 to 7. Line graph shows curve-fitted data from three independent experiments. (**g** and **h**) RT112 cultures were preincubated with Chol (10 μg/ml) for 6 h before the addition of mTORi (0.25 μm; **g**) or AKTi (1 μm; **h**) and CQ (1–40 μm) for 5 days. Histograms show the quantification of cell death from four independent experiments. Statistical analysis on blue (mTORi+CQ) or red (AKTi+CQ) bars reflects comparison of means to CQ control (black). Statistics on gold bars (+Chol) reflects significance to red and blue bars at each concentration of CQ (i.e. protection by Chol against combination treatment) (**i**). MGH-U3 cells were treated and immunoblotted as in (**a**). (**j**) MGH-U3 cells were treated as indicated and cell death quantified and curve-fitted to generate EC50 values (data from one experiment is shown; *n*=3; statistical analysis by Student's *t*-test on data at 10 μm CQ). *P<0.05, **P<0.01, ***P<0.001.

Immunoblotting shows that FGFRi and mTORi fully suppress the expression of cSREBP1 and cholesterol enzymes in RT112 cultures, whereas AKTi and PI3Kαi partially suppress this pathway (Figures 4a and b). Remarkably, suppression of cSREBP1 by FGFR/PTENi significantly correlates with the exacerbation CQ-induced cell death; Pearson's *r* (*R*^2^)=0.871 (*P*=0.0065; Figures 4b–c). Thus, CQ-induced cell death is not modulated by PI3Kβi (*P*=0.9657 to ctrl), but similarly exacerbated by PI3Kαi (*P*=0.0008 to ctrl) and AKTi (*P*=0.0002 to ctrl; *P*=0.9013 to PI3Kαi). However, inhibition of mTOR (*P*<0.0001 to ctrl) and FGFR (*P*=0.0003 to ctrl) further potentiate CQ-induced cell death, although the curve for FGFRi is clearly different to the other conditions, suggesting interference by additional factors. To further investigate cholesterol biosynthesis in CQ-induced LCD, we inhibited HMGCR—a rate-limiting enzyme in cholesterol biosynthesis—with Ato, or saturated cellular membranes with water-soluble cholesterol.^36, 37^ Figure 4d shows that Ato exacerbates CQ-induced cell death, whereas cholesterol is significantly protective. Furthermore, we could recapitulate the effects of AKTi on CQ toxicity by knocking down the expression of HMGCS1 or DHCR7 (Figures 4e and f).

Importantly, at low doses of CQ, a clear synergy is observed with mTORi and AKTi, which is robustly (mTORi) or completely (AKTi) suppressed by cholesterol (Figures 4g and h). However, cholesterol did not protect against the monotherapy toxicity of either FGFR or mTORi, nor other apoptotic insults including staurosporine, TRAIL (tumour necrosis factor-related apoptosis-inducing ligand) and proteasome inhibition, showing that it is not generally protective against apoptosis (Supplementary Figures S4B and C). Furthermore, cholesterol did not inhibit lysosomal neutralization by CQ or BafA1, demonstrating that protection is conferred downstream of the function of these compounds (Supplementary Figures S4D and E). Suppression of cSREBP1 and cholesterol biosynthesis enzymes by FGFRi, mTORi and AKTi was also confirmed in the other type-III line, MGH-U3, wherein cholesterol again significantly protects against synergistic cell death induced by CQ and AKTi (Figures 4i and j). However, MGH-U3 cultures do not show suppression of cSREBP1 or potentiation of CQ-induced cell death with PI3Kα inhibition, likely due to the expression of a downstream AKT1(E17K)-activating mutation, which sustains AKT activation and PRAS40 phosphorylation under PI3Kαi. Thus, suppression of cSREBP1 and cholesterol biosynthesis pathway correlates with the potentiation of CQ-induced cell death, which is robustly inhibited by saturation of cellular membranes with cholesterol.

### Cholesterol depletion by AKTi sensitises lysosomes to CQ-induced membrane permeabilisation

**Figure 5:**
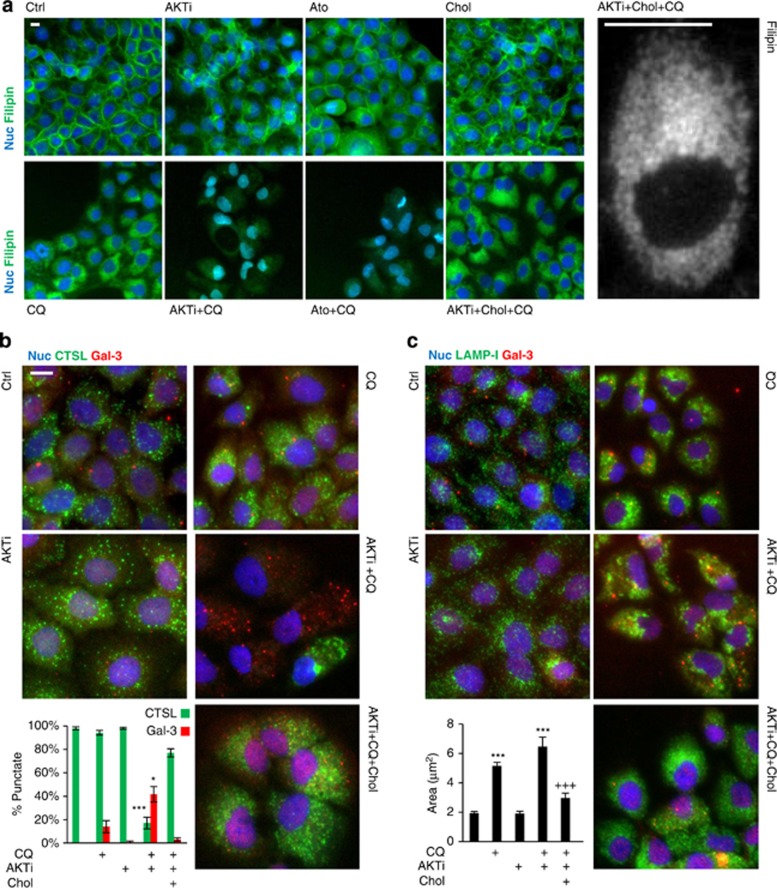
Water-soluble cholesterol saturates intracellular membranes under CQ treatment and rescues loss of cathepsin release, lysosomal damage and swelling. (**a**) RT112 cells were treated with AKTi (1 μM), Ato (5 μM), Chol (10 μg/mL) and CQ (20 μM) as indicated for 3 days, fixed and stained with filipin to visualise cholesterol (green; Hoechst staining in blue; *n*=3). High-magnification imaging reveals that Chol saturates numerous punctate vesicle-like structures throughout the cytosol, consistent with endo/lysosomal localisation (right). (**b** and **c**) RT112 cells were treated as in (**a**) and stained for cathepsin L (CTSL; green, **b**), LAMP-I (green, **c**) and galectin-3 (Gal-3; red; **b** and **c**). Representative immunomicrographs are shown alongside the quantification of punctate CTSL/Gal-3 staining (**b**; *n*=3) and lysosomal area (**c**; *n*=3; *P<0.05, ***P<0.001 to control, ^+++^*P*<0.001 to CQ+AKTi). Scale bars represent 10 μM.

We used filipin to visualise cellular cholesterol and show that whereas AKTi and Ato alone cause little measureable change to cellular cholesterol, both compounds combine with CQ to deplete severely intracellular cholesterol (Figure 5a). Under these conditions, cholesterol treatment rescues cellular cholesterol, saturating intracellular vesicle-like structures throughout the cell. These observations were also quantified by fluorometric detection of free cellular cholesterol (Supplementary Figure S4F). To assay lysosomal membrane permeabilisation, we quantified the loss of cathepsin immunoreactivity from punctate cytosolic structures and the formation of galectin-3 foci, a marker for damaged endomembranes.^38^ Figure 5b shows that the combination of CQ with AKTi results in the substantial loss of punctate cathepsin staining and a significant increase in galectin-3 aggregates. Remarkably, these changes were almost completely prevented by cholesterol. Furthermore, lysosomal swelling induced by CQ alone or in combination with AKTi is also robustly blocked by cholesterol (Figure 5c). However, we could not detect the cytosolic translocation of lysosomal cathepsins by microscopy or fractionation (indicating a short half-life once released). Nevertheless, we found that the combination of CQ with AKTi causes lysosomal cathepsin depletion, correlating with cytochrome *c* release, before the onset of caspase activation (Supplementary Figure S4G). Taken together, our data show that cellular cholesterol depletion and lysosomotropic stress by AKTi+CQ causes lysosomal swelling, damage, cathepsin release and cell death, which are profoundly rescued by saturating cellular membranes with cholesterol.

### Cholesterol availability is a general determinant to CQ sensitivity

**Figure 6:**
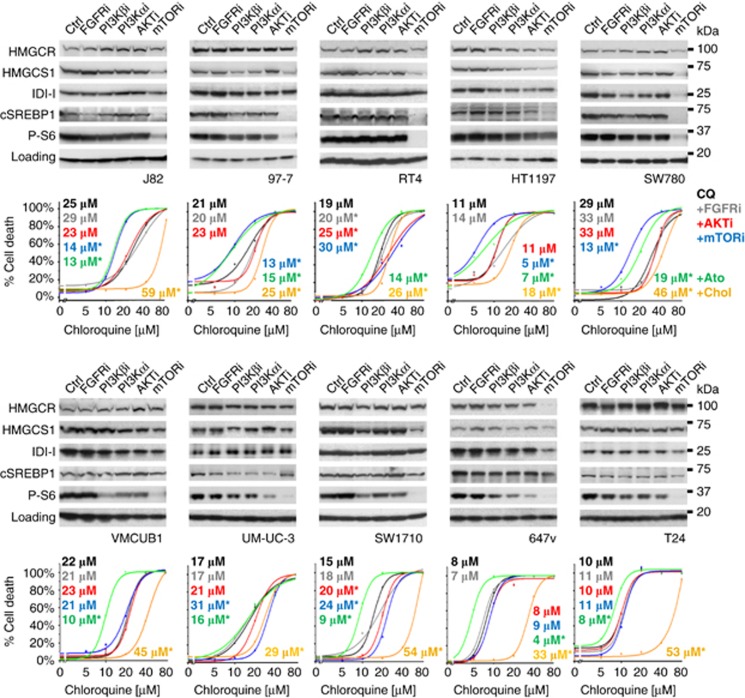
Potentiation of CQ-induced cell death is linked to the suppression of cSREBP1 expression and recapitulated with atorvastatin (Ato), or inhibited by water-soluble cholesterol (Chol), across the bladder cell line panel. Bladder cancer cell lines carrying FGFR3 mutations (top row), or WT for FGFR3 (bottom row), were treated as indicated (1 μm) for 5 days before lysis and immunoblotting for the indicated proteins (*n*=2). Line graphs show curve-fitted cell death quantifications in response to CQ alone (black lines; 0–80 μm) or in combination with FGFRi (grey), AKTi (red), mTORi (blue), Ato (green) or Chol (gold; data from one representative experiment is shown; *n*=3). EC50 values are shown inset with corresponding indication of statistical significance (*n*⩾3; *P<0.05 indicates where the difference between treatment and control means reaches significance at any concentration of CQ).

Figure 6 shows that type-I and -II bladder cancer cell lines do not show the same regulation of SREBP1 by FGFRi, PI3Ki and AKTi as type-III cells (RT112 and MGH-U3), despite the presence (top) or absence (bottom) of FGFR3 mutations. However, all cell lines carrying FGFR3 mutations showed suppression of cSREBP1 expression by mTORi, which correlated with a significant exacerbation of CQ-induced cell death in four out of five cell lines. All cell lines showed robust protection against CQ toxicity with cholesterol, whereas Ato exacerbated cell death. We also found that cholesterol significantly protects against BafA1-induced cholesterol loss, cell death and lysosomal swelling across the bladder panel (Supplementary Figures S5A–D). However, Ato did not potentiate BafA1 toxicity, indicating that maximal cell death is elicited at normal cellular cholesterol concentrations; a feature that mitigates its use as a therapeutic (Supplementary Figure S5E).^39^ Moreover, cholesterol significantly protects against both CQ and BafA1 cytotoxicity in primary human fibroblasts, suggesting that a widespread mode of cell death induced by these compounds is lysosomal destabilisation, as opposed to the inhibition of autophagy (Supplementary Figure S5F).^40, 41, 42, 43^ Overall, this study indicates a general role for cholesterol homeostasis in maintaining lysosomal membrane integrity under stress, which supports a rationale for combinations of CQ with targeted therapeutics that block cholesterol metabolism in cancer cells.

## Discussion

Despite the increasing number of reports suggesting a role for autophagy in the response of cancers to therapeutics, little detail has emerged to account for the molecular mechanisms that elicit either prosurvival or prodeath autophagy.^9^ Nevertheless, CQ is currently being used as an adjuvant to anticancer therapies in over 30 clinical trials.^9^ Herein, we have shown across a genetically diverse range of bladder cancer lines that synergy between mTOR pathway inhibition and CQ is restricted to FGFR3-mutant cancers, and that suppression of cholesterol metabolism, not autophagy, underlies lysosomal destabilisation and cell death.

### Role of autophagy in bladder cancer

Our knockdown of numerous autophagy-targeting genes convincingly demonstrates that the activation of autophagy does not contribute to the innate resistance of bladder cancers to FGFR/PTENi. These experiments highlight the differential requirements for autophagy under genuine starvation, where autophagy inhibition exacerbates cell death, compared with inhibitors of mitogenic signalling, where suppression of autophagy does not enhance cell death. Our data do however support a role for autophagy in the optimal growth of bladder cancer cells, which may underlie the trend for autophagy siRNAs or *ATG7* KO to mildly protect against FGFRi and mTORi.^44, 45^ Furthermore, by robustly blocking autophagosome degradation and recycling with CQ and BafA1, we also rule out a role for constitutive bulk autophagic flux as a cytoprotective pathway under AKTi and mTORi in bladder cancers. These experiments did, however, identify a synergy between CQ and AKT or mTOR inhibitors in FGFR-dependent cancers. Our subsequent work brings together recent findings describing the regulation of cholesterol biosynthesis by FGFR3 and the function of cholesterol in regulating LCD.^34, 35, 43^

### Modulation of CQ-induced cell death by inhibition of cholesterol biosynthesis

The induction of LCD by CQ has been well characterised in HeLa cells and recently shown to be potentiated by a dual PI3K/AKT inhibitor in neuroblastomas.^29, 30, 46, 47^ Our model shares significant similarities with these reports, as synergistic cell death is unrelated to autophagy and requires cathepsin and BID activity upstream of caspase activation. However, we further show that the degree to which FGFR/PTENi potentiate CQ cytotoxicity correlates with suppression of cSREBP1 and loss of cellular cholesterol. Importantly, both FGFRi and mTORi lower the EC50 of CQ to therapeutically relevant concentrations (~6 μm), indicating that CQ may considerably improve the efficacy of these compounds *in vivo*.^42, 48, 49^

The finding that PI3Kαi or AKTi partially suppresses cSREBP1 expression, whereas FGFRi and mTORi completely suppress this pathway, suggests that FGFR regulates mTORC1/2-cSREBP1 through both PI3K/AKT-dependent and -independent (for example, PLCγ or RAS/MAPK) signalling. This is consistent with reports that SREBP1 is regulated by multiple pathways and indicates that the complete suppression of cSREBP1 is required to maximally potentiate CQ-induced cell death.^50, 51, 52^ Moreover, we found that all FGFR3-mutant cell lines stabilise cSREBP1 via mTORC1/2, yet do not show the same regulation of cSREBP1, or potentiation of CQ-induced cell death, with FGFRi or AKTi (Figure 6). Since S6 phosphorylation is also maintained under these conditions, we interpret this data to indicate that other mTORC1/2 signalling pathways are activated in these cells, which are sufficient to promote SREBP1 activation in the FGFR3 mutant background. Interestingly, the presence of FGFR3 mutations correlates with the potentiation of CQ-induced cell death in only six of the seven cell lines. RT4 cells do not fit this trend, possibly due to a coincident RhoA(A161V) mutation that may alter cholesterol or membrane dynamics.^53^ In contrast, FGFR3-WT cell lines regulate cSREBP1 expression independently of mTORC1/2 and therefore do not show potentiation of CQ-induced cell death with mTORi. Rather, many of these lines show significant protection against CQ toxicity with mTORi, suggesting that whether mTORi potentiates or inhibits CQ-induced cell death may be determined by the balance of its effects on cholesterol availability versus inhibition of proliferation.

As activating FGFR3 mutations are found in up to 80% of low-grade noninvasive bladder tumours, the combination of CQ with mTOR pathway inhibitors may therefore benefit a substantial population of patients.^26^ Other transforming RTK mutations may similarly control cholesterol biosynthesis and present opportunities for eliciting synergistic cell death between relevant inhibitors and CQ. We provide proof of principle for this approach by showing that inhibition of cholesterol metabolism with a statin, or knockdown of key cholesterol biosynthesis enzymes, recapitulates synergistic cell death in numerous cell lines. However, in RT112 cells, knockdown of HMGCS1 and DHCR7, or Ato treatment, exacerbated cell death to a similar extent as AKTi, rather than to the level observed with mTORi. Additionally, the suppression of cSREBP1 by mTORi in FGFR3-mutant lines only partially inhibited the expression of cholesterol enzymes, yet resulted in a similar potentiation of CQ toxicity to Ato. Conversely, some FGFR3-WT cells showed mTORi-induced suppression of cholesterol enzyme expression in the absence of changes to cSREBP1 expression and CQ-induced cell death. Thus, additional mechanisms may regulate the expression of these enzymes and the availability of cellular cholesterol, which will require further elucidation.

### Role of cholesterol in regulating lysosomal membrane integrity

**Figure 7:**
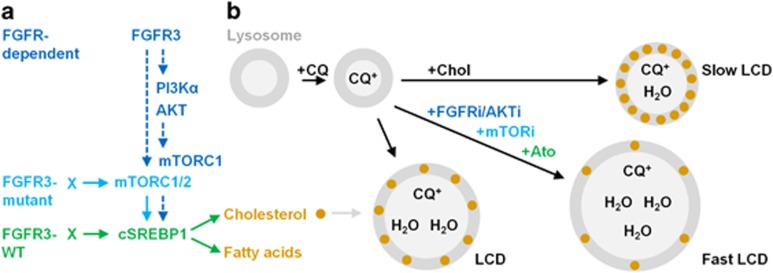
FGFR signalling and model for the role of cholesterol biosynthesis in the maintenance of lysosomal membrane integrity under CQ- and BafA1-induced stress. (**a**) FGFR-dependent (blue text) and FGFR3-mutant (light blue text only) bladder cancer cell lines maintain cSREBP1 expression in an mTORC1/2-dependent manner (left). In contrast, FGFR3-WT cells (green text) maintain cSREBP1 independently of mTORC1/2 signalling. (**b**) CQ is a weak base that diffuses into lysosomes (grey circles), neutralises the pH and becomes trapped by protonation, inducing osmotic stress, lysosomal swelling, cathepsin release and lysosomal cell death (LCD). Inhibition of FGFR, AKT or mTOR signalling in FGFR-dependent cell lines or HMGCR (Ato) in all cell lines, depletes cellular cholesterol and accelerates LCD (Fast LCD). In contrast, saturation of cellular membranes with water-soluble cholesterol (+Chol) prevents CQ-induced lysosomal swelling, cathepsin loss and cell death (Slow LCD). Lysosomal membrane cholesterol is known to regulate permeability to water and ions; however, this is the first study to demonstrate that inhibition of cholesterol biosynthesis underlies the potentiation of CQ-induced LCD in cancer cells. Furthermore, we propose that water-soluble cholesterol can be used to determine the relative contribution of LCD versus autophagy inhibition in future studies examining the potentiation of therapeutic-induced cell death with lysosomal inhibitors.

Our data conclusively demonstrate a key role for cholesterol biosynthesis in regulating lysosomal integrity and cell death under lysosomotropic stress. These results can be explained by known features of cholesterol homeostasis and lysosomal membrane biology: (i) neutralisation of the endo/lysosomal system blocks the processing of extracellularly derived cholesterol esters, rendering cells dependent on the biosynthesis pathway; and (ii) lysosomal membrane cholesterol decreases permeability to water and ions, suppressing swelling and destabilization under osmotic stress.^54, 55, 56^ Thus, under inhibition of both cholesterol uptake (for example, CQ) and cholesterol biosynthesis (for example, AKTi), osmotic stress is enhanced, resulting in accelerated membrane permeabilisation, cathepsin release and cell death (Figure 7; blue text).^57^ In contrast, saturation of cellular membranes with cholesterol blocks CQ- and BafA1-induced LCD upstream of swelling and membrane permeabilisation. Given the efficacy by which water-soluble cholesterol prevents both BafA1- and CQ-induced LCD, we propose that this method can be used as a novel assay to clarify the contribution of LCD versus autophagy inhibition in cell death studies combining CQ with other therapeutics *in vitro*.^38^

### Concluding remarks

Our results delineate for the first time the mechanistic basis for synergy between CQ and a targeted anticancer therapeutic and stress caution in the interpretation of studies reporting enhanced drug efficacy in combination with CQ. These data also highlight the exciting therapeutic potential for CQ in combination with mTOR pathway inhibitors in patients with activating FGFR3 mutations. Finally, by revealing a role for cholesterol biosynthesis in maintaining lysosomal integrity under stress, we propose that therapeutics which suppress cholesterol metabolism in other cancer types may present similar opportunities for eliciting synergistic cell death with CQ, thereby defining a novel approach to future cancer treatments.

## Materials and methods

### Cell culture

Reagents were obtained from Sigma (St Louis, MO, USA), except where indicated. Cell lines were sourced from: RT112/84 (ECACC, Salisbury, UK); TCC-SUP, J82, SW780, T24, 1A6, UM-UC-3, HT-1197, HT-1376, 5637, RT4, SCaBER (ATCC, Manassas, VA, USA); VM-CUB-1, 647-V, BFTC-905, KU-19-19, SW1710 (DSMZ, Braunschweig, Germany); MGH-U3 and 97-7 (LIMM, Leeds, UK); and human fibroblasts (Promocell, Heidelberg, Germany). All cell lines tested mycoplasma negative by Chan test. Cells were cultured in DMEM (Dulbecco's modified Eagle's medium) containing 10% fetal calf serum and 1% l-glutamine at 37 °C (5% CO_2_) and passaged weekly. For nutrient starvation, cells were washed three times in prewarmed EBSS (Gibco, Grand Island, NY, USA). AZD4547, AZD8186, AZD8835, AZD5363, AZD2014 and Ato were made by AstraZeneca (Macclesfield, UK) and resuspended in dimethyl sulfoxide to 10 mm. All other reagents were reconstituted in dimethyl sulfoxide except: CQ (200 mm phosphate-buffered saline (PBS)); 3MA (20 mm DMEM); cycloheximide (10 mg/ml water), cholesterol (10 mg/ml PBS); and *N*-acetyl-l-cysteine (100 mm DMEM). CA-074Me was obtained from Calbiochem (San Diego, CA, USA). Free cholesterol was quantified using ab65359 Kit (Abcam, Cambridge, UK). SmartPOOL ON-TARGET siRNAs (Dharmacon, Lafayette, CO, USA) were resuspended in water at 10 μm and reverse transfected by adding nine volumes of cell suspension to one volume of siRNA (10 nm final concentration) premixed with Lipofectamine RNAiMAX (Invitrogen, Carlsbad, CA, USA) in OPTI-MEM (Gibco).

### Microscopy and cell death assays

Cells were seeded at a density of 5 000/ml in 96-well plates and stained with SYTOX Green Nucleic Acid Stain (Molecular Probes, Eugene, OR, USA) and Hoechst 33342 (1 μg/ml) following treatment. Total and dead cells were quantified using a Cellomics ArrayScan XTI (ArrayScan; 12 fields; >1000 cells) with appropriate filter settings. cdEC50s were calculated using Origin 7.5 SR6 (OriginLab Corporation, Northampton, MA, USA). For CASP3 activation assays, NucView-488 Caspase-3 substrate (Biotum, Hayward, CA, USA) was incubated with cells at 1 μm in an IncuCyte ZOOM (Essen Bioscience, Ann Arbor, MI, USA) for 5 days. The frequency of NucView-488 signals was quantified using ZOOM GUI version 2014A, and normalised for cell confluency using processing definitions adapted for each cell line.

### Immunocytochemistry

Cells were seeded in 96-well plates at a density of 0.5–5x10^4^/ml and formalin-fixed for 20 min following treatment. Cultures were washed three times in PBS and blocked (3% bovine serum albumin, 0.1% Triton) for 1 h before overnight incubation (4 °C) with primary antibodies and 1 h incubations with secondary antibodies. For LC3 and LAMP-I immunostaining, cultures were postfixed in 100% ice-cold methanol for 15 min. Antibodies were purchased from Cell Signalling Technologies (Danvers, MA, USA) except: anti-galectin-3 (BD Biosciences, San Jose, CA, USA), anti-cathepsin L (Abcam). Secondary antibodies from Molecular Probes were used at a 1:500 and visualised by ArrayScan. LAMP-I puncta were quantified using ImageJ (NIH, Bethesda, MD, USA) by measuring the area of the three largest and clearly defined immunopositive structures per cell (three fields; three independent experiments). Galectin-3-positive cells were defined by the presence of three or more cytosolic aggregates. For filipin staining, fixed cultures were quenched in glycine (1.5 mg/ml PBS; 10 min), rinsed and incubated with 25 μg/ml filipin-PBS (60 min) washed and visualised by ArrayScan.

### Immunoblotting

Cells were seeded at 0.5–2x10^5^/ml and lysed in sodium dodecyl sulphate-based buffer following treatment. Lysates were normalised for protein concentration (BCA assay; Pierce, Rockford, IL, USA), boiled in sample buffer (NuPage+DTT; Life Technologies, Grand Island, NY, USA) and loaded at 10 μg per lane on Criterion gels (4–20%). Gels were transferred to nitrocellulose, blocked (5% milk-TBS-T) and incubated overnight (4 °C) with primary antibodies. All antibodies were from Cell Signaling Technology except: cathepsin B (Calbiochem), HMGCS1 (Sigma), FRS-2 (R&D Systems) and HMGCR, IDI-I and DHCR7 (Abcam). Horseradish peroxidase-conjugated secondary antibodies were incubated for 1 h and visualised by ECL (Perkin-Elmer, Waltham, MA, USA) using film (GE Healthcare, Uppsala, Sweden) or CCD (Chemigenius; Syngene, Frederick, MD, USA). Films were scanned at 600 dpi and analysed for densitometry using ImageJ (NIH). Cellular fractionation was performed using Kit No. 9038 from Cell Signalling.

### CRISPR

A sequence targeting ATG7 exon 1 (5′-AAGCTGAACGAGTATCGGC-3′) was synthesised (Sigma) within a guide RNA plasmid also encoding Cas9-GFP. The Cas9/gRNA was purified by Endo-free maxi prep (Qiagen, Germantown, MD, USA), transfected using FuGENE HD (Promega, Fitchburg, WI, USA). Cells were sorted for GFP fluorescence after 48 h (BD FACSAria Ilu, BD Biosciences) and seeded at 1 cell per well in 96-well plates. Cultures were monitored for single clones until confluency, passaged and assayed for ATG7 and LC3-II expression by western blotting.

### Statistics

Data were analysed for statistical significance using one-way analysis of variance followed by Dunnett's *post hoc* test to compare treatment means to control means (JMP12.0.1; SAS, Cary, NC, USA). Where indicated, Student's *t*-test was used to compare means from at least three independent experiments.
