# Supplementary figures and images for: Inhibition of cholesterol metabolism underlies synergy between mTOR pathway inhibition and chloroquine in bladder cancer cells
Source: Oncogene. 2016 Feb 8;35(34):4518–28. doi: 10.1038/onc.2015.511 (PMC5000518; doi:10.1038/onc.2015.511)

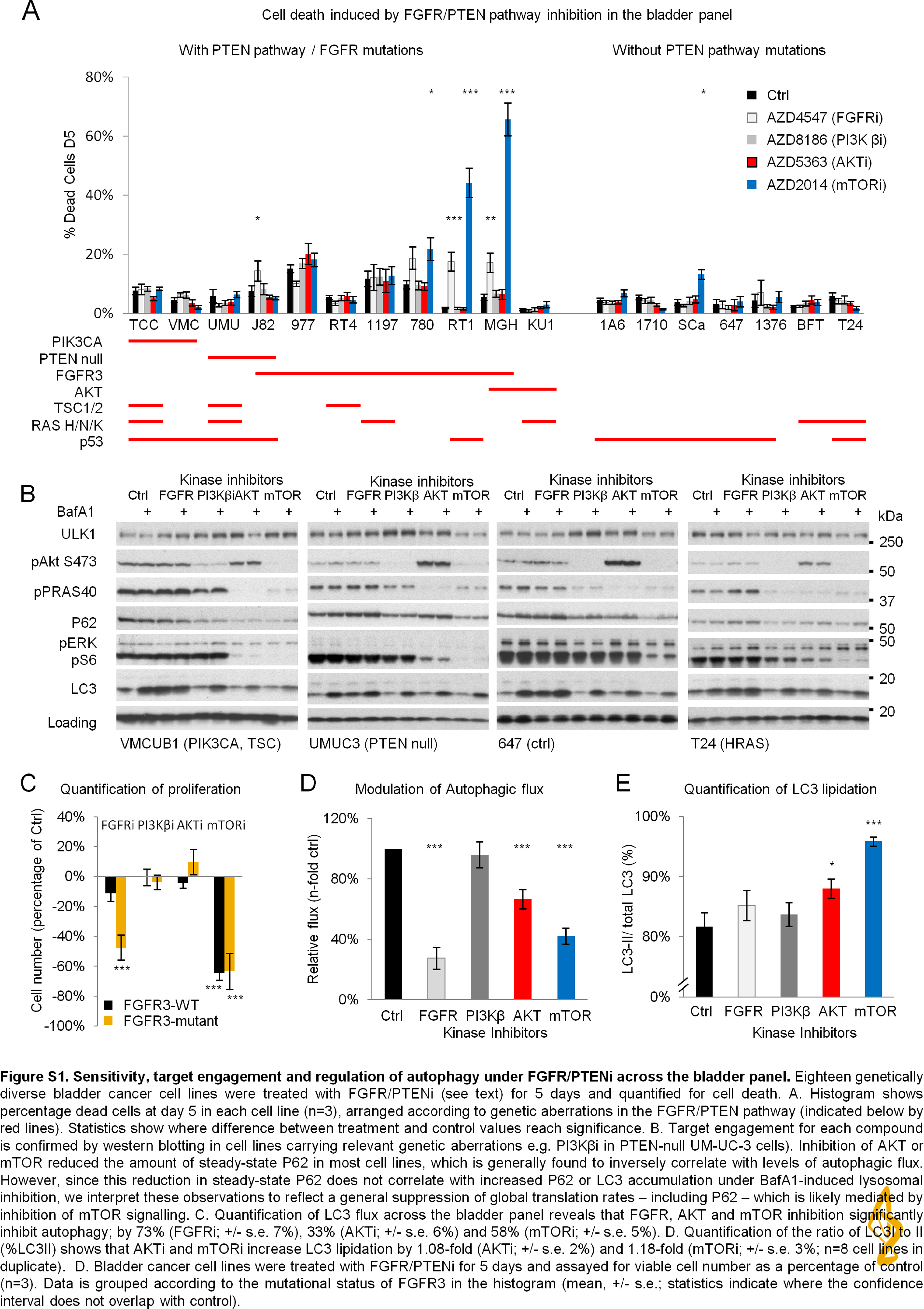

Supplement: Supplementary Figure S1 [file onc2015511x1.tif]

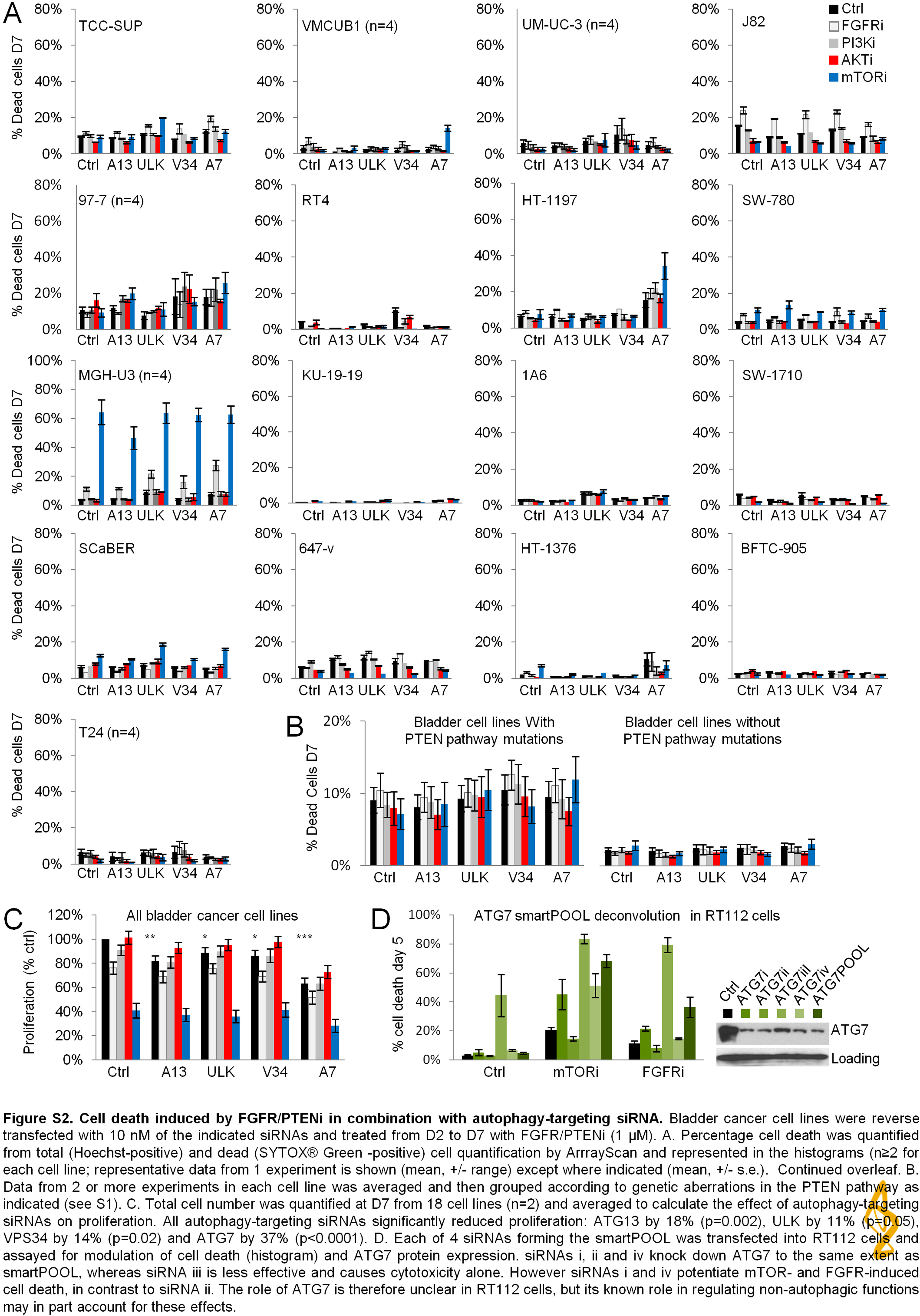

Supplement: Supplementary Figure S2 [file onc2015511x2.tif]

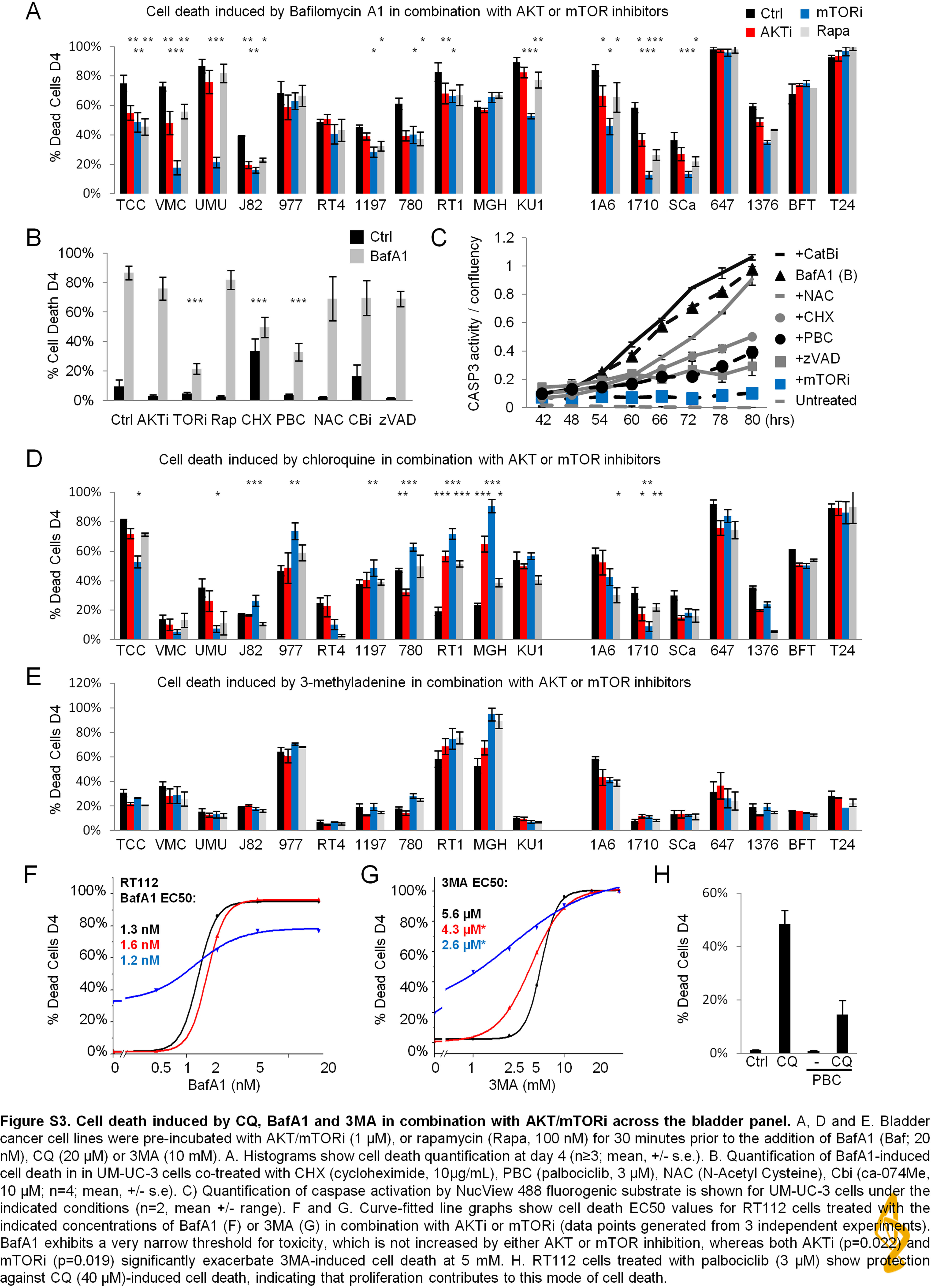

Supplement: Supplementary Figure S3 [file onc2015511x3.tif]

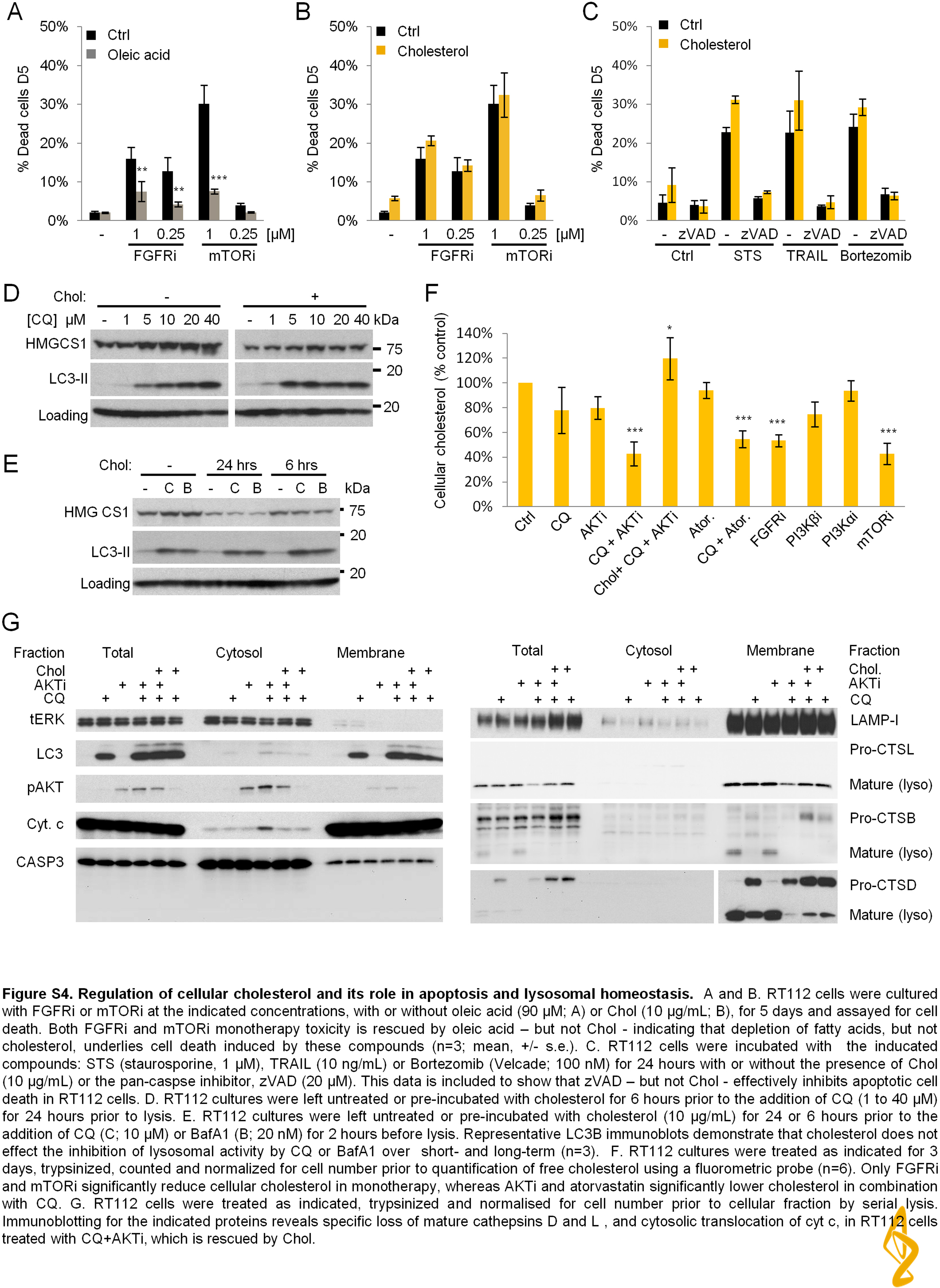

Supplement: Supplementary Figure S4 [file onc2015511x4.tif]

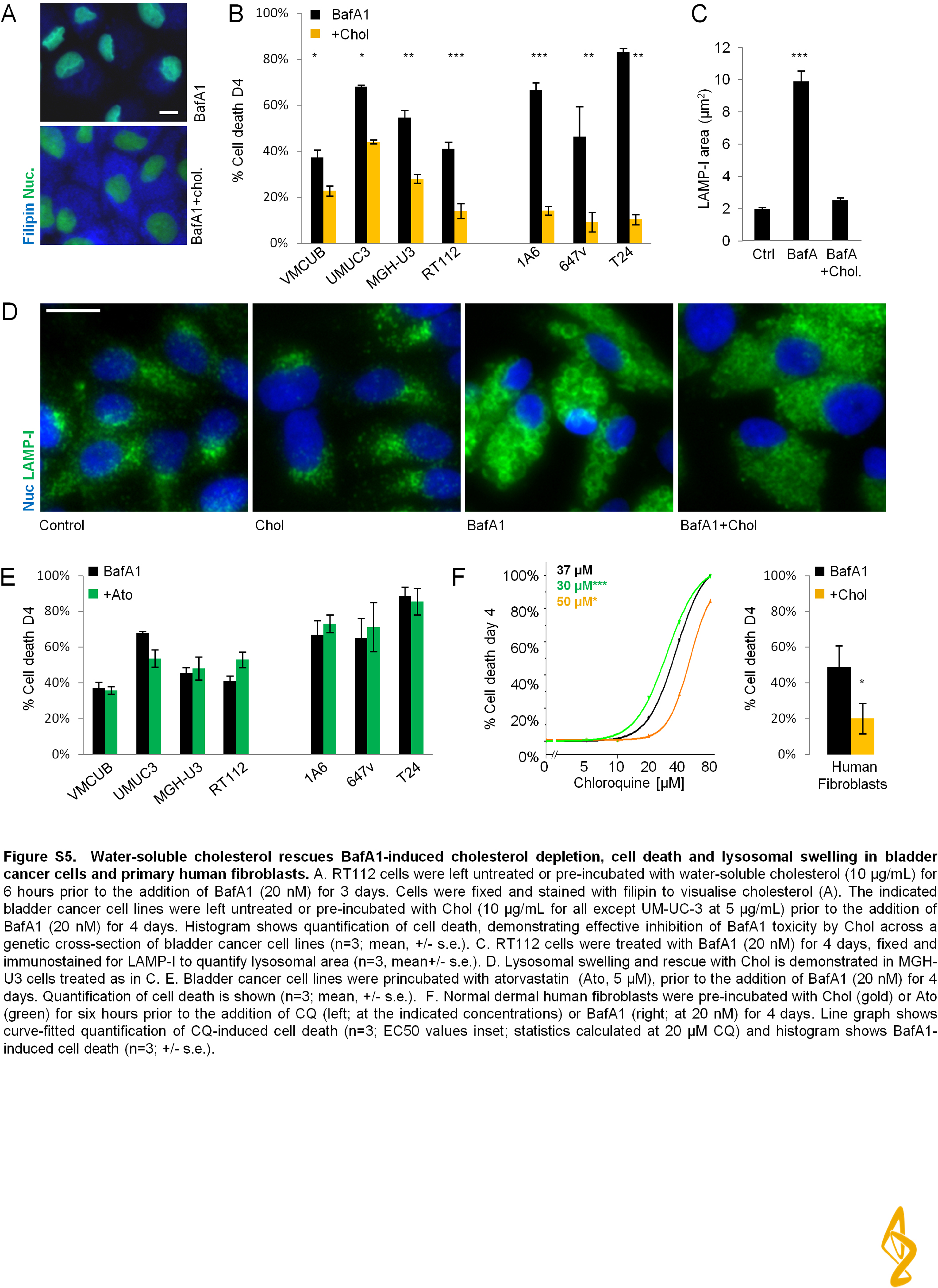

Supplement: Supplementary Figure S5 [file onc2015511x5.tif]
